# A Longitudinal Study of Subjective Daytime Sleepiness Changes in Elementary School Children Following a Temporary School Closure Due to COVID-19

**DOI:** 10.3390/children8030183

**Published:** 2021-03-01

**Authors:** Yoko Komada, Yoshiki Ishibashi, Shunta Hagiwara, Mariko Kobori, Akiyoshi Shimura

**Affiliations:** 1 Faculty of Liberal Arts, Meiji Pharmaceutical University, 2-522-1 Noshio, Kiyose, Tokyo 204-8588, Japan; 2 Department of Preventive Medicine and Public Health, Keio University School of Medicine, 35 Shinanomachi, Shinjuku-ku, Tokyo 160-8582, Japan; ishiyoshi414@gmail.com; 3 Graduate School of Economics, The University of Tokyo, 7-3-1, Hongo, Bunkyo-ku, Tokyo 113-0033, Japan; shunta.hagiwara@gmail.com; 4 Department of Pharmacy and Health Sciences, Meiji Pharmaceutical University, 2-522-1 Noshio, Kiyose, Tokyo 204-8588, Japan; y171124@std.my-pharm.ac.jp; 5 Department of Psychiatry, Tokyo Medical University, 6-7-1 Nishi-Shinkuku, Shinjuku, Tokyo 160-0023, Japan; sim@tokyo-med.ac.jp

**Keywords:** children, sleepiness, Pediatric Daytime Sleepiness Scale (PDSS), later school start times, school closure, COVID-19, midpoint of sleep, midsleep, social jetlag

## Abstract

Excessive daytime sleepiness is increasingly being recognized as a major global health concern. However, there have been few studies related to sleepiness and its associated factors in elementary school children. In Japan, all schools were closed from February to May 2020 to prevent coronavirus disease 2019 (COVID-19) outbreaks. The aim of this study was to identify changes in the subjective sleepiness of pupils during the 1.5-year period and to elucidate factors associated with changes in sleepiness. Questionnaire surveys about pupils’ sleep habits and the Japanese version of the Pediatric Daytime Sleepiness Scale (PDSS-J) were conducted longitudinally at one elementary school in June 2019, January 2020, and June 2020. The average ∆PDSS score was 0.94 ± 5.51 (mean ± standard deviation) from June 2019 to January 2020 and −1.65 ± 5.71 (t[498] = 6.13, *p* < 0.01) from January 2020 to June 2020. Univariate and multivariate logistic regression analyses revealed that decreasing social jetlag was associated with decreasing PDSS scores (OR = 0.77, 95% CI: 0.62–0.96, *p* = 0.02) during the school closure. A less restrictive school schedule secondary to a COVID-19-related school closure decreased sleepiness in children and was associated with decreasing social jetlag.

## 1. Introduction

Daytime sleepiness increases significantly with age during adolescence [1]. Sleep laboratory experiments using the Multiple Sleep Latency Test (MSLT) across different age groups have revealed a tendency for greater sleepiness in the mid-afternoon than in the morning or evening in pubertal adolescents [1]. Studies using the Pediatric Daytime Sleepiness Scale (PDSS), a self-rated measurement of daytime sleepiness, have shown that this score increases with age [2,3]. The prevalence of excessive daytime sleepiness (PDSS > 18) increased from 19.8% at prepuberty to 47.2% at post-puberty [4]. Thus, it has been demonstrated that objective and subjective sleepiness increase concurrently with pubertal maturation, while the capacity for sustained daytime alertness in younger children.

Excessive daytime sleepiness is increasingly being recognized as a major global health concern, affecting up to 40% of children and adolescents [5,6]. Notably, the bedtimes of Asian children and adolescents are later than those of their peers in North America or Europe, resulting in less total sleep time and higher daytime sleepiness [7].

There have been several studies of associated factors with daytime sleepiness in adolescents. Short sleep duration [8,9,10,11], late chronotype [12], sleep disturbances [13], low stress control [11], and lifestyle factors such as electronics use [14,15] are associated with daytime sleepiness. A meta-analysis examining the effects of school start times on sleep and daytime sleepiness has revealed that later school start times induce longer sleep durations and less daytime sleepiness in adolescents [16]. However, there have been few studies related to sleepiness and its associated factors in elementary school children.

In Japan, all schools were closed from February to May 2020 to prevent COVID-19 outbreaks [17]. During this period, pupils studied at home according to their schedules. We conducted a longitudinal survey of sleep habits and PDSS scores at an elementary school in Japan at the three survey points. The aims of this study were (1) to investigate whether pupils’ subjective sleepiness changed during the 1.5-year period, especially before the school closure (June 2019 and January 2020) and just after reopening of schools (June 2020) and (2) to elucidate factors associated with changes in sleepiness.

## 2. Materials and Methods

All study procedures were conducted following the guidelines outlined in the Declaration of Helsinki. The ethical review board of the Neuropsychiatric Research Institute in Japan approved the study protocol (No. 0205).

The study was conducted longitudinally at one elementary school in Tokyo, Japan. The survey was conducted as part of a biannual health check-up in June 2019, January 2020, and June 2020. The school comprised a total of 652 pupils in grades 1 through 6 in June 2019, 651 pupils in January 2020, and 687 pupils in June 2020. Informed consent was obtained from the pupils’ parents. The questionnaire was delivered to pupils by their schoolteacher and answered in the classroom. For younger children (grades 1 and 2, aged 6–7 years), the teacher assisted with questionnaire completion by explaining the meaning of words and how to answer the questions. After the questionnaires were collected by teachers, the school nurse recorded the heights and weights of pupils.

The numbers of pupils who answered the questionnaire in June 2019, January 2020, and June 2020 were 619 (94.9%), 609 (93.5%), and 646 (94.0%), respectively (Table 1). The percentage of boys was 47.3% in 2019 and 46.9% in 2020.

This questionnaire surveyed pupils about their bedtimes, wake-up times, at-home study times, screen-viewing times on weekdays and weekends, and daytime sleepiness during the past month. Daytime sleepiness during the past month was also assessed using the Japanese version of the Pediatric Daytime Sleepiness Scale (PDSS-J) [2,3]. The PDSS is an 8-item self-report that asks children about their experiences of sleepiness. Items include ‘How often do you fall asleep or feel drowsy in class?’ and ‘Are you usually alert during the day?’ Total scores can range from 0 to 32, with higher scores indicating more daytime sleepiness. The PDSS showed adequate internal consistency for the data in the current study, with a Cronbach’s alpha of 0.80 across grades 1–6 (0.75 in 1st and 2nd grades, 0.83 in 3rd and 4th grades, and 0.80 in 5th and 6th grades).

The following variables were calculated for each pupil: sleep duration and mid-point of sleep (midsleep) were calculated from using bedtimes and wake-up times. Social jetlag, an assessment of circadian misalignment, was calculated as the difference between mid-sleep on weekends and midsleep on weekdays [18]. To quantify the magnitude of changes in these variables, we calculated individual deltas (∆) for each parameter for the periods from January 2020 to June 2019 and from June 2020 to January 2020.

Data from the 513 pupils who answered all three surveys were used for longitudinal analyses. Grade 1 pupils in 2020 and grade 6 pupils in 2019 were excluded, as we could not obtain longitudinal data owing to admission or graduation during the study period (Table 1).

### Statistical Analysis

PDSS scores were compared cross-sectionally between grades at each survey point (June 2019, January 2020, and June 2020) using an analysis of variance (ANOVA) followed by the Bonferroni-Dunn’s post-hoc test. The six grades were categorized into lower (1st and 2nd grades, aged 6–7 years), middle (3rd and 4th grades, aged 8–9 years), and upper (5th and 6th grades, aged 10–11 years) grades.

Next, sleep variables were compared longitudinally between the three survey points using a repeated measures ANOVA and Bonferroni–Dunn’s post-hoc test. The distributions of ∆PDSS scores were analyzed separately at January 2020 and June 2020 and categorized into the following three groups: a PDSS score increase of more than 3 points from January 2020 to June 2020 (∆PDSS score), a PDSS score decrease of more than 3 points, or a PDSS score change of between −3 and 3 points. Changes in sleep variables were compared between the three groups using ANOVA and Bonferroni–Dunn’s post-hoc test.

Factors associated with a decline in daytime sleepiness were examined using a series of logistic regression analyses. All variables were initially examined in univariate models. Then, to control for confounding factors and to determine main correlates, multivariate logistic regression analysis was conducted for variables, accounting for multicollinearity.

Statistical analyses were performed using SPSS version 22 (SPSS Inc., Chicago, IL, USA) with the level of significance set at a *p*-value of < 0.05.

## 3. Results

### 3.1. Comparison of PDSS Scores in the Lower, Middle, and Upper Grades

The six grades were categorized into lower (1st and 2nd grades), middle (3rd and 4th grades), and upper (5th and 6th grades) grades, and PDSS scores were compared between these categories at each survey point (Table 2). There were significant differences in PDSS scores between these categories at the different survey points (June 2019: F[2, 611] = 9.0, *p* < 0.01, January 2020: F[2, 606] = 8.2, *p* < 0.01, and June 2020: F[2, 644] = 3.8, *p* = 0.02). The Bonferroni–Dunn’s post-hoc test revealed that PDSS scores in the upper grades were significantly higher than PDSS scores in the lower or middle grades, while there were no significant differences in PDSS scores between the lower and middle grades.

For sleep time on weekdays, the means and standard deviations (SDs) in the lower, middle, and upper grades were 9:41 ± 1:01, 9:21 ± 0:58, and 9:36 ± 1:06, respectively, in June 2020 (F[2, 640] = 19.8, *p* < 0.01); 9:35 ± 0:35, 9:15 ± 0:55, and 8:50 ± 0:53, respectively, in January 2020 (F[2, 531] = 33.7, *p* < 0.01); and 9:32 ± 0:38, 9:16 ± 0:54, and 8:46 ± 0:48, respectively, in June 2019 (F[2, 528] = 39.7, *p* < 0.01).

### 3.2. Comparison of Sleep Habits and Other Variables between the Three Survey Points

Sleep habits, at-home study times, and screen-viewing times were investigated in pupils who had answered the questionnaire at all three survey points (Table 3). Repeated measures ANOVA showed significant differences in weekday bedtimes (F[2, 1012] = 27.0, *p* < 0.001), weekday wake-up times (F[2, 1010] = 48.2, *p* < 0.001), midsleep on weekdays (F[2, 1002] = 59.9, *p* < 0.001), weekend bedtimes (F[2, 996] = 10.0, *p* < 0.001), weekend wake-up times (F[2, 982] = 7.2, *p* < 0.001), and midsleep on weekends (F[2, 970] = 14.7, *p* < 0.001). Post-hoc tests revealed that bedtimes, wake-up times, and midsleep on weekdays were significantly delayed as the survey timepoints progressed (June 2019 < January 2020 < June 2020). There were no significant differences in bedtimes, wake-up times, or midsleep on weekends between the January 2020 survey and the June 2020 survey; however, these variables were significantly delayed in the 2020 surveys compared to the 2019 survey (June 2019 < January 2020, June 2019 < June 2020). The total sleep time on weekdays was significantly longer in the June 2020 survey than in the June 2019 or January 2020 surveys (F[2, 1002] = 4.0, *p* = 0.02), while there were no significant differences in total sleep times on weekends between the three surveys. The relative value of social jetlag was significantly smaller in June 2020 than in June 2019 or January 2020 (F[2, 968] = 5.2, *p* < 0.01).

At-home study times and screen-viewing times on both weekdays and weekends were significantly longer in June 2020 than in January 2020 or June 2019 (at-home study time on weekdays: F[2, 964] = 150.8, *p* < 0.001; at-home study time on weekends: F[2, 846] = 44.7, *p* < 0.001; screen-viewing time on weekdays: F[2, 880] = 64.5, *p* < 0.001; screen-viewing time on weekends: F[2, 938] = 19.7, *p* < 0.001).

### 3.3. Longitudinal Changes in Daytime Sleepiness

**Figure 1:**
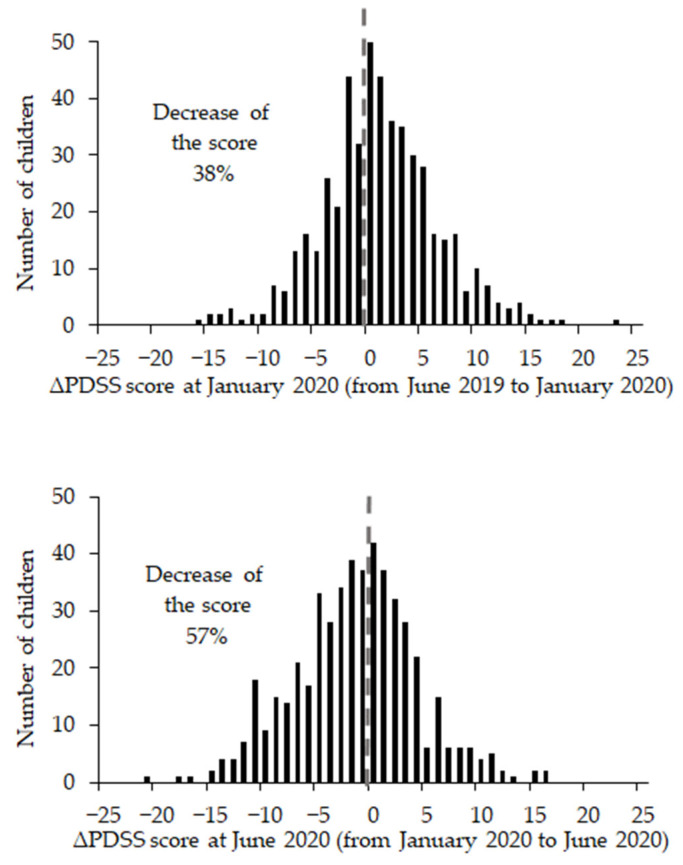
The distribution of ∆PDSS scores at January 2020 and June 2020.

The ∆PDSS score from June 2019 to January 2020 was 0.94 ± 5.51, while the ∆PDSS score from January 2020 to June 2020 was −1.65 ± 5.71. There was a significant difference in the ∆PDSS score between the January 2020 and June 2020 survey points (t[498] = 6.13, *p* < 0.01). Figure 1 shows the distribution of ∆PDSS scores. The percentage of pupils whose PDSS score decreased between survey points was 38.1% between June 2019 and January 2020 and 56.9% between January 2020 and June 2020.

### 3.4. The Relationship between Changes in Sleep Habits and Daytime Sleepiness

**Figure 2:**
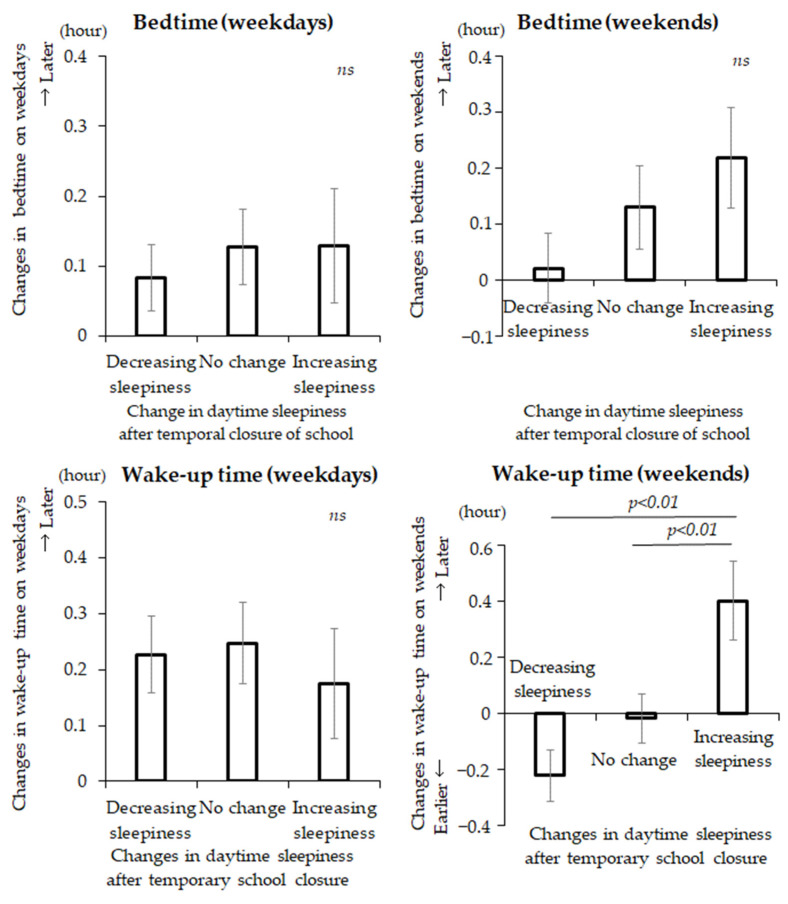

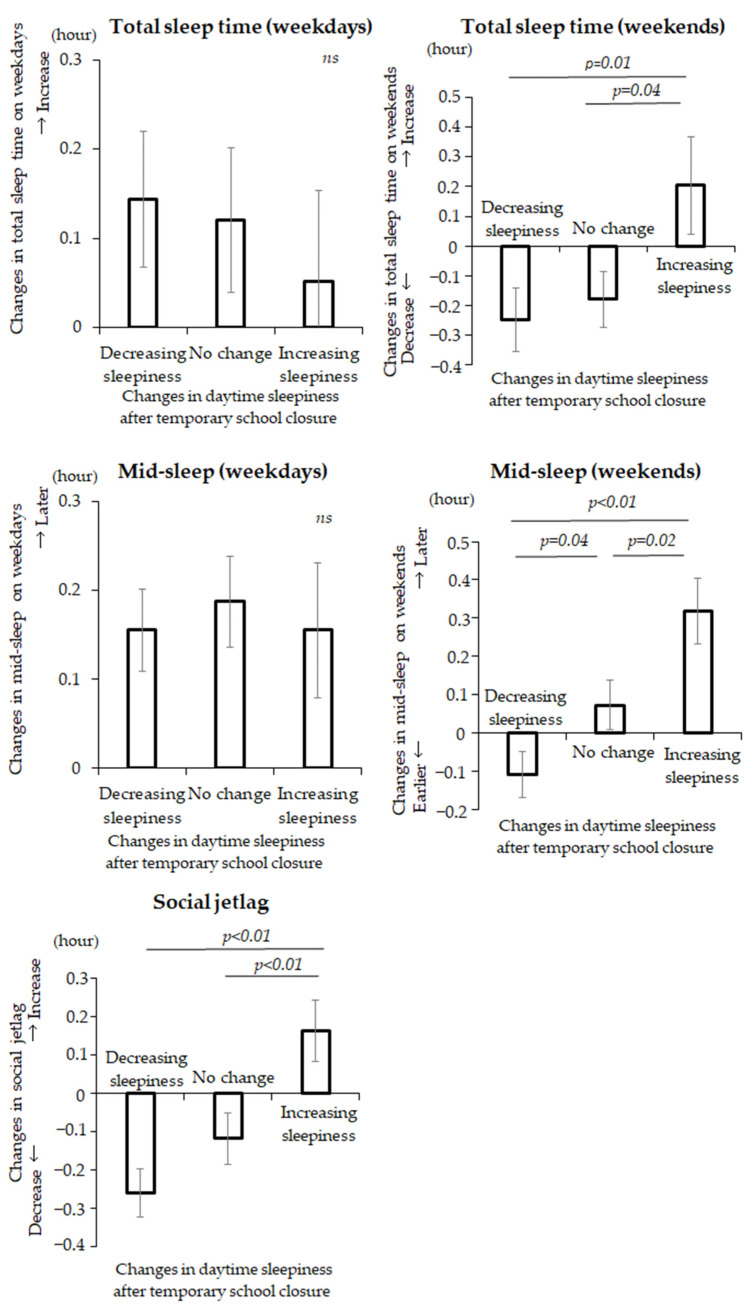
Comparison of changes in sleep variables between three groups categorized by their daytime sleepiness changes (∆PDSS score) from January to June 2020.

Pupils were categorized into three groups according to the changes in their daytime sleepiness (∆PDSS score) between January 2020 and June 2020, including a decreasing-sleepiness group, a no-change group, and an increasing-sleepiness group. Changes in other sleep variables were then compared for these three groups over the same period. Figure 2 shows a comparison of the changes in sleep variables in the three groups. An ANOVA revealed that there were no significant differences in weekday sleep variable changes between the three groups (bedtime on weekdays: F[2, 493] = 0.22, *p* = 0.81; wake-up time on weekdays: F[2, 495] = 0.18, *p* = 0.84; midsleep on weekdays: F[2, 492] = 0.12, *p* = 0.89; total sleep time on weekdays: F[2, 492] = 0.25, *p* = 0.78). In contrast, there were significant differences in weekend sleep variables changes (wake-up time on weekends: F[2, 489] = 8.0, *p* < 0.01; midsleep on weekends: F[2, 485] = 8.4, *p* < 0.01; and total sleep time on weekends: F[2, 485] = 3.4, *p* = 0.04). The Bonferroni-Dunn’s post-hoc test revealed that the ∆wake-up time and the ∆midsleep on weekends were significantly delayed, and the ∆total sleep time on weekends was lengthened from January to June 2020 in the increasing-sleepiness group compared with the decreasing-sleepiness group and the no-change group. The increasing-sleepiness group also showed a significantly increasing ∆social jetlag from January to June 2020 compared to the decreasing-sleepiness and no-change groups (F[2, 484] = 7.9, *p* < 0.01).

There were no significant differences in the ∆study time at home on weekdays (F[2, 473] = 2.7, *p* = 0.07) or on weekends (F[2, 413] = 1.8, *p* = 0.16) or in the screen-viewing time on weekdays (F[2, 451] = 0.58, *p* = 0.56) or on weekends (F[2, 466] = 0.44, *p* = 0.64) between the three groups.

### 3.5. Factors Associated with a Decline in Daytime Sleepiness

The factors associated with decreasing PDSS scores were examined. Univariate logistic regression analyses were performed for the following independent variables: sex, grade, body mass index (BMI), sleep variables, at-home study time, and screen-viewing time on both weekdays and weekends. Among these variables, four items (advancing wake-up time on weekends, advancing midsleep on weekends, decreasing total sleep time on weekends, and decreasing social jetlag) exhibited significant correlations with decreasing PDSS scores. Adjusted odds ratios (ORs) and their 95% confidence intervals (CIs) were as follows: wake-up time on weekends: OR = 0.76, 95% CI: 0.66–0.88, *p* < 0.01; midsleep on weekends: OR = 0.67, 95% CI: 0.54–0.83, *p* < 0.01; total sleep time on weekends: OR = 0.86, 95% CI: 0.76–0.97, *p* = 0.02; and social jetlag: OR = 0.73, 95% CI: 0.59–0.90, *p* < 0.01. The other variables (sex, grade, BMI, bedtime, and wake-up times on weekdays, total sleep time on weekdays, bedtime on weekends, at-home study time on weekdays and weekends, and screen-viewing time on weekdays and weekends) were not significantly associated with decreasing PDSS scores. Multivariate logistic regression analysis, accounting for multicollinearity, was then conducted, using the variables of grade, total sleep time on weekends, and social jetlag. Decreasing social jetlag (OR = 0.77, 95% CI: 0.62–0.96, *p* = 0.02) was significantly associated with decreasing subjective daytime sleepiness, while total sleep time on weekends showed a trend association (OR = 0.90, 95% CI: 0.79–1.0, *p* = 0.09).

## 4. Discussion

The present study examined changes in subjective daytime sleepiness in elementary school students. After determining the PDSS scores cross-sectionally, we found that PDSS scores were higher in the upper grades (5th and 6th grades, aged 10–11 years) than in the lower (1st and 2nd grades, aged 6–7 years) or middle (3rd and 4th grades, aged 8–9 years) grades, without a significant difference between the lower and middle grades. These results suggest that subjective sleepiness increases at around 10 years of age, which is the timepoint at which children typically enter secondary sexual development. Next, the data from the three survey points were longitudinally investigated. The PDSS score changed from 9.6 ± 6.2 points in June 2019 to 10.5 ± 6.2 points in January 2020 (∆PDSS: 0.94 ± 5.51), whereas it changed from 10.5 ± 6.2 points in January 2020 to 8.9 ± 6.3 points in June 2020 (∆PDSS: −1.65 ± 5.71). These results indicate that subjective daytime sleepiness was decreased in pupils during the COVID-19-related school closure.

Before discussing these findings further, it is important to address the issue of whether the PDSS score is a valid reflection of daytime sleepiness in elementary school children. The validity of this measure has been confirmed in middle school children aged 11–15 years [2], and it is known to be a suitable tool for evaluating child sleepiness [3,19,20]. Although the PDSS has not been specifically validated in samples younger than 11 years of age, there is no reason to question its ability to detect sleepiness in children of school age (>5 years), provided that the individual has sufficient reading skills [2]. In this study, Cronbach’s alpha ranged from 0.75 for lower-grade pupils to 0.83 for middle-grade pupils. Overall, Cronbach’s alpha was 0.80, which indicates an acceptable consistency.

The average PDSS score was significantly increased during the 6 months from June 2019 to January 2020. This study did not check the Tanner stage for each pupil; therefore, we could not group participants into prepubertal versus post-pubertal categories. However, children of this age are typically in the process of developing toward adolescence. Therefore, it has been suggested that increasing daytime sleepiness in children of this age results from biological changes, including a decline in delta wave power during nocturnal sleep and hormonal changes [1,21,22].

In contrast, the subjective sleepiness of pupils decreased from January to June 2020, with more than half of pupils showing a decrease in PDSS scores over that period. One explanation for this decrease may be the COVID-19-related school closure, with fewer restrictions on school schedules. Of note, the group with increasing daytime sleepiness during this period had significantly later sleep–wake phases on weekends, longer sleep times on weekends, and greater social jetlag than either the decreasing-sleepiness group or the no-change group. These results suggest that daytime function deteriorated when the sleep–wake phase became further delayed due to the school closure. On the other hand, we speculate that the daytime function of pupils can be enhanced if sleep-wake schedules are concordant with chronotypes.

The American Academy of Pediatrics has recommended that junior high and high schools should delay school start times to 08:30 h or later [23] to prevent insufficient sleep, which can result in daytime dysfunction. Several previous studies have demonstrated that delaying school start times is an effective measure to prevent chronic sleep loss, with a wide range of potential benefits for physical and mental health, safety, and academic achievement [8,24,25]. In contrast, similar studies in elementary school children are scarce, possibly because the effects of later school start times are greater in junior high and high school students, since most adolescents begin to experience a sleep-wake phase delay manifested as a shift of up to 2 h relative to the sleep-wake cycle of middle childhood [26,27].

Rather than manipulating school start times, the present study examined changes in the subjective sleepiness of elementary school children during a COVID-19-related school closure. Therefore, our results cannot conclusively state whether school start times should be delayed for elementary schools. However, the finding that subjective sleepiness was decreased in 57% of pupils under the less restrictive school schedule, with less social jetlag, certainly provides material for further discussion of school start times. A study of school start times in elementary school children in Boston indicated that a change to an earlier school start time did not decrease the total sleep time [28]. Furthermore, it did not increase sleep debt, as evidenced by weekend bedtimes and wake-up times, as well as self-reported sleepiness during or after school [28]. In that study, the average sleep duration of the middle grades at baseline (before the change in school start time) was more than 10 h (10:11 in 3rd grade, 10:03 in 4th grade) [28]. In contrast, the average sleep duration for the same grades in our study was 9:15 before the school closure, indicating that the sleep duration in Japanese pupils is nearly 1 h less than their counterparts in the US. The baseline sleep debt of these Japanese pupils is thought to have influenced changes in sleep habits and daytime sleepiness during the temporary school closure. That is, prepubertal elementary school children can intrinsically maintain high awareness throughout the day if they get the necessary amount of sleep [1]; however, if pupils get less than the 10 h of sleep recommended by the National Sleep Foundation for children aged 6–13 years [29], daytime sleepiness can occur, even in prepubertal children. Further research is needed on the relationship between daytime sleepiness, sleep habits, and school schedules in elementary school children.

The logistic regression analysis showed that decreasing subjective daytime sleepiness during the school closure was associated with decreasing amount of catch-up sleep on weekends and less social jetlag. The results suggest that the alignment of weekend and weekday sleep patterns secondary to less restrictive school schedules may lead to improved daytime function. Of note, subjective sleepiness was increased in pupils with delayed sleep-wake phases and increased social jetlag under the less restrictive school schedule. Therefore, it should be emphasized that keeping regular sleep-wake schedule and preventing social jetlag is important under the less restrictive school schedule including school closure, later or flexible school start times. A randomized controlled trial of sleep extension vs. no intervention for adolescents with chronic sleep loss indicated that adolescents in the sleep-extension group had earlier sleep onsets, more time in bed, and slept longer than adolescents in the control group during the third week of the intervention [30]. In addition, insomnia and depressive symptoms were significantly diminished in the sleep-extension group. These findings suggest that a gradually advancing bedtime and a gradual sleep extension in combination with sleep hygiene advice to develop a more regular sleep schedule can have beneficial effects on sleep problems and depressive symptoms in adolescents. We could not provide any sleep hygiene advice for pupils during the school closure; however, this study suggests that it is necessary for elementary pupils with chronic sleep loss in our country to not only to get enough sleep but also to avoid social jetlag for improvement of daytime function.

This study had some limitations. First, administration of this questionnaire to pupils at only one elementary school limits the generalizability of our findings to other settings. However, we did conduct our longitudinal survey before (January 2020) and after (June 2020) COVID-19-related school closures and during an ordinary situation (June 2019), and we obtained high response rates. Second, the present preliminary study could not account for the widespread and complicated influence of the pandemic on children’s health and welfare, and these factors need to be fully considered in future research. Third, we did not obtain an objective evaluation of sleep in our study; however, the PDSS is widely used to assess sleepiness in children. Furthermore, we were able to carry out our research without risk to the students, even during the COVID-19 pandemic.

## 5. Conclusions

Subjective daytime sleepiness in elementary school children was investigated longitudinally using the Japanese version of the PDSS. Subjective daytime sleepiness increased with increasing grade levels. A less restrictive school schedule secondary to a COVID-19-related school closure decreased sleepiness in children and was associated with decreasing social jetlag.

## Figures and Tables

**Table 1:** Number of pupils and respondents at each survey point.

June 2019					January 2020					June 2020				N ^1^
G ^2^	P ^3^	R ^4^	C ^5^		G ^2^	P ^3^	R ^4^	C ^5^		G ^2^	P ^3^	R ^4^	C ^5^	
Before admission				→	Before admission				→	1st	113	111	98.2	
1st	136	135	99.3	→	1st	136	131	96.3	→	2nd	137	132	96.4	128
2nd	103	100	97.1	→	2nd	103	98	95.1	→	3rd	106	99	93.4	96
3rd	116	104	89.7	→	3rd	116	106	91.4	→	4th	119	105	88.2	96
4th	100	91	91.0	→	4th	100	91	91.0	→	5th	102	94	92.2	89
5th	110	107	97.3	→	5th	110	106	96.4	→	6th	110	105	95.5	104
6th	87	82	94.3	→	6th	86	77	89.5	→	After graduation				
Total *n*, %	652	619	94.9			651	609	93.5			687	646	94.0	513

^1^ Number of respondents at all three surveys points *n*; ^2^ Grade (1st: 6 years old to 6th: 11 years old); ^3^ Pupils *n*; ^4^ Respondents *n*; ^5^ Collection rate %.

**Table 2:** Cross-sectional comparison of PDSS scores between grades.

Grade ^1^ Survey Point	Lower (L) Mean SD		Middle (M) Mean SD		Upper (U) Mean SD		*p Value* ^2^	*Post Hoc* ^3^
June 2019	8.9	5.4	8.9	6.4	11.2	6.6	*<0.001*	L < U **, M < U **
January 2020	9.7	5.7	10.0	6.5	12.0	6.2	*<0.001*	L < U **, M < U **
June 2020	8.2	5.4	7.9	6.5	9.5	6.1	*<0.02*	L < U *, M < U **

^1^ Lower grade: 1st and 2nd grades, Middle grade: 3rd and 4th grades, Upper grade: 5th and 6th grades; ^2^ Result of analysis of variance (ANOVA); ^3^ Results of the Bonferroni–Dunn’s post-hoc test was used to compare PDSS score among grades. **: *p* < 0.01, *: *p* < 0.05.

**Table 3:** Comparison of sleep habits and other variables at the three survey points in pupils who responded to all three surveys.

	June 2019 ^a^ Mean SD		January 2020 ^b^ Mean SD		June 2020 ^c^ Mean SD		*p Value* ^1^	*Post Hoc* ^2^
Bedtime on weekdays	21:28	0:53	21:37	0:52	21:43	0:57	*<0.0001*	a < b < c **
Bedtime on weekends	21:52	1:06	21:58	1:05	22:04	1:03	*<0.0001*	a < b,c **
Wake-up time on weekdays	6:37	0:34	6:47	0:33	7:00	1:01	*<0.0001*	a < b<c **
Wake-up time on weekends	7:25	1:21	7:37	1:15	7:37	1:22	*<0.001*	a < b,c **
Total sleep time on weekdays	9:09	0:51	9:10	0:52	9:16	1:04	*0.02*	a,b < c *
Total sleep time on weekends	9:35	1:18	9:40	1:15	9:32	1:20	*0.11*	
Midsleep on weekdays	2:03	0:36	2:12	0:35	2:22	0:50	*<0.001*	a < b<c **
Midsleep on weekends	2:38	1:02	2:47	0:59	2:51	1:01	*<0.001*	a < b,c **
Social jetlag (relative value)	0:35	0:45	0:36	0:45	0:29	0:47	*0.01*	c < a *, c < b **
Social jetlag (absolute value)	0:43	0:37	0:44	0:37	0:41	0:38	*0.27*	
PDSS score ^3^	9.6	6.2	10.5	6.2	8.9	6.3	*<0.001*	c < a < b **
At-home study times on weekdays (min)	42.2	51.9	46.0	55.4	100.8	107.3	*<0.0001*	a,b < c **
At-home study times on weekends (min)	52.1	87.5	62.1	95.4	90.0	114.6	*<0.0001*	a,b < c **; a < b *
Screen-viewing on weekdays (min)	94.9	95.7	99.1	88.1	161.2	162.8	*<0.0001*	a,b < c **
Screen-viewing on weekends (min)	143.5	161.5	154.6	147.5	198.1	208.4	*<0.0001*	a,b < c **

^1^ Results of repeated analysis of variance (ANOVA); ^2^ Results of Bonferroni-Dunn, ^a^: June 2019, ^b^: January 2020, ^c^: June 2020, **: *p* < 0.01, *: *p* < 0.05; ^3^ PDSS: Pediatric Daytime Sleepiness Scale.

## Data Availability

The data presented in this study are available on request from the corresponding author. The data are not publicly available due to ethical restriction.
